# Predictors of Middle School Students' Interest in Participating in an Incentive-Based Tobacco Prevention and Cessation Program in Connecticut

**DOI:** 10.1155/2014/915652

**Published:** 2014-07-24

**Authors:** Meghan E. Morean, Deepa R. Camenga, Grace Kong, Dana A. Cavallo, Ty S. Schepis, Suchitra Krishnan-Sarin

**Affiliations:** ^1^Department of Psychiatry, Yale School of Medicine, CMHC, 34 Park Street, New Haven, CT 06519, USA; ^2^Department of Pediatrics, Yale School of Medicine, 330 Cedar Street LMP 4086, New Haven, CT 06520, USA; ^3^Department of Psychology, Texas State University, 601 University Drive, San Marcos, TX 78666, USA

## Abstract

Behavioral incentives have been used to encourage smoking cessation in older adolescents, but the acceptability of incentives to promote a smoke-free lifestyle in younger adolescents is unknown. To inform the development of novel, effective, school-based interventions for youth, we assessed middle school students' interest in participating in an incentive-based tobacco abstinence program. We surveyed 988 students (grades 6–8) attending three Connecticut middle schools to determine whether interest in program participation varied as a function of (1) intrapersonal factors (i.e., demographic characteristics (sex, age, race), smoking history, and trait impulsivity) and/or (2) aspects of program design (i.e., prize type, value, and reward frequency). Primary analyses were conducted using multiple regression. A majority of students (61.8%) reported interest in program participation. Interest did not vary by gender, smoking risk status, or offering cash prizes. However, younger students, non-Caucasian students, behaviorally impulsive students, and students with higher levels of self-regulation were more likely to report interest. Inexpensive awards (e.g., video games) offered monthly motivated program interest. In sum, middle school students reported high levels of interest in an incentive-based program to encourage a tobacco-free lifestyle. These formative data can inform the design of effective, incentive-based smoking cessation and prevention programs in middle schools.

## 1. Introduction

Tobacco use is the leading cause of preventable morbidity and mortality in the United States [1, 2]. According to the National Youth Tobacco Survey [3], 5% of middle school students (grades 6–8) currently smoke cigarettes and 20% of nonsmokers report willingness to experiment with cigarettes. A growing body of research indicates that trait impulsivity, or “a predisposition toward rapid, unplanned action…with diminished regard to negative consequences” [4] may further increase risk for smoking initiation [5–7]. High levels of intent to smoke among young adolescents may be driven in part by intrapersonal factors like impulsivity and contribute to sharp increases in smoking uptake among high school students. Unfortunately, early initiation of smoking predicts future regular smoking and increases the likelihood of developing lifetime respiratory conditions, cardiovascular disease, and cancers [8]. Thus, effective tobacco prevention/intervention programs in middle schools are needed.

Unfortunately, few well-designed prevention or intervention trials have shown promise in middle schools [9]; successful programs include the prevention-focused “Project Towards No Tobacco Use” [10] and the cessation-focused “Not on Tobacco” [11], and Project Ex [12]. Furthermore, even successful programs are plagued by low participation rates [13]. Improving program effectiveness depends on our ability to employ innovative methods to increase participation and retention.

Incentive-based programs, which provide rewards (e.g., money/prizes) in exchange for confirmed tobacco abstinence, have promise for application in middle schools. Prior research indicates that community-based “Quit and Win” programs, which enroll smokers into contests wherein they may win prizes for being tobacco-free, are most appealing to younger smokers [14]. Behavioral reinforcement effectively prompts smoking cessation in treatment-seeking adults [15, 16], high school students [17, 18], and at the community level [19]. Recent research also indicates that incentive-based programs may be particularly effective for impulsive adolescent smokers [20] who struggle disproportionately to quit smoking [21]. With respect to impulsivity, children and adolescents demonstrate normative deficits in planning for the future and exhibit high discount rates relative to older adolescents and adults [22]. Research suggests that cigarette smoking (and even experimentation) may exacerbate these deficits; adolescent smokers/experimenters have more difficulty planning for the future, exhibit higher discount rates, and self-report higher levels of impulsivity relative to adolescent nonsmokers [23]. As mentioned previously, impulsive adolescent smokers also are less likely to successfully quit smoking after participating in a cessation program than their less impulsive counterparts [21]. When considering the prior research findings in concert, an incentive-based approach may be ideal to encourage smoking cessation (and perhaps prevent smoking uptake) among a wide range of adolescents, especially those with higher impulsivity.

Although self-determination theory [24, 25] argues that financial incentives may undermie intrinsic motivation to sustain behavior change, a recent review of the psychology and economics literature concluded that the evidence is not sufficient to conclude that motivation for health-related behaviros would be adversely affected by incentives [26]. Rather, incentives may enhance self-control or ability to refuse tempting behaviors (such as smoking), therefore enhancing perceived competence while preserving (if not boosting) intrinsic motivation [26].

With regard to evaluating the effectiveness of classroom-based, behavioral incentive programs, a recent meta-analysis of “The Smoke-Free Class” competition [27], a program implemented in over 20 European countries that rewards classes for being smoke-free at 6-month intervals, found that contest participation decreased the likelihood of smoking initiation. However, selection bias may have inflated program efficacy; not all classes enrolled in the program. To maximize the effectiveness of future incentive-based programs, it is essential that we understand the factors that increase or decrease program enrollment.

To our knowledge, no incentive-based smoking prevention/cessation programs have been developed for use with middle schools in the United States. To inform the development of such a program, the current study evaluated the extent to which intrapersonal factors (i.e., demographics, tobacco use status, and trait impulsivity) and program-design features (i.e., prize type, value, and frequency of reward) influence students' interest in participating.

Given the low participation rates of smokers in existing tobacco programs, we hypothesized that smokers and individuals deemed susceptible to smoking would be less interested than nonsusceptible nonsmokers. Although there are clear links between impulsivity and smoking, it is unclear whether impulsive adolescents would be more or less interested in* participating* in an incentive-based prevention/intervention program. Impulsive adolescents may be less interested if they perceive the rewards to be neither immediate nor guaranteed and lack the foresight to appreciate the long-term health benefits of living smoke-free. However, impulsive adolescents may be more interested in participating if the prospect of receiving a reward is reinforcing in itself and is perceived to be proximal relative to the distal experience of future health benefits [26]. Therefore, we did not outline a specific hypothesis regarding the relationship between impulsivity and program interest. Lastly, we hypothesized that offering inexpensive rewards valued at $25 would motivate student interest based on the age range of our sample (11–14 years). To contextualize this monetary value, adolescents ages 16–18 spend an average of only $18 per week [28]. We believe that the current study findings can inform the development of future, successful incentive-based programs designed to encourage tobacco-free living in middle school students.

## 2. Materials and Methods

We obtained approval from the Yale Institutional Review Board and school officials at three Connecticut middle schools prior to administering a brief, anonymous, cross-sectional, self-report survey of students' tobacco use. Two-weeks before the survey, we also mailed information sheets to all students' parents/guardians. Parents were told to call the research staff or school officials if they did not want their child to participate (no parent explicitly refused permission). Otherwise, passive parental permission was assumed. These methods have been used successfully in similar school-based work [29].

Prior to survey completion, students were informed of the limits to confidentiality and the voluntary nature of the study and, subsequently, provided assent. Surveys were administered in a general assembly and comprised 25 questions assessing demographic characteristics, tobacco use, and students' views on the incentive-based program.

### 2.1. Participants

Nine hundred eighty-eight adolescents completed the survey (50.1% female, mean age 12.41 [SD = 1.02] years, 69.8% Caucasian, 5.0% lifetime smokers, 10.0% susceptible to smoking).

### 2.2. Measures

#### 2.2.1. Program Interest

The following description of an incentive-based program was provided within the survey: “We are interested in developing a program that would encourage middle school students to stop using tobacco products and to prevent students who do not use from ever starting. This program would be open to all students, whether or not they currently use tobacco. It involves a chance to enter a contest and make a pledge to not use any tobacco products. Anyone who is tobacco free at the end of the contest period would have a chance to win a prize.” We assessed students' interest in participating using the following question: “How interested would you be in participating in the contest?” Response options included not at all, probably not, might or might not be interested, probably interested, and definitely interested. In total, 61.8% of students reported that they would probably or definitely be interested in participating in the contest.

#### 2.2.2. Intrapersonal Measures

*Demographics.* We assessed gender, age, and race/ethnicity.

*Cigarette Smoking Status.* Smoking status was assessed with the following item from the Monitoring the Future Survey and National Survey on Drug Use and Health: “Do you currently use, or have you ever used, any of the following tobacco products? (cigarettes, cigars, smokeless tobacco, bidis, cloves, or I never tried any tobacco products). Students who reported ever using cigarettes were classified as lifetime smokers (*n* = 43). Nonsmokers were classified using the Fishbein/Ajzen-Hanson questionnaire [30] as either susceptible to initiating smoking (*n* = 86) or not susceptible (*n* = 681) based on self-reported intention to smoke (i.e., “I intend to smoke a month from now” was assessed three separate times with the following 7-point anchors: (1) extremely likely to extremely unlikely, (2) extremely probably to extremely not probably, and (3) extremely true to extremely untrue). Students were classified as low-risk nonsmokers only if they selected all three indicators that they had no intent to smoke (i.e., extremely unlikely, extremely not probably, and extremely untrue). All other nonsmokers were considered susceptible to smoking. Given the low prevalence of smoking in the current sample, lifetime smokers and students considered to be at risk for initiating cigarette smoking were combined into a single high-risk category.

*Impulsivity.* Students completed the 30-item Barratt Impulsiveness Scale, Version 11 [31], which assesses a range of impulsive and nonimpulsive (reverse scored) behaviors using a 4-point scale (response options include rarely/never, occasionally, often, and almost always/always). A large body of research suggests that the 3 originally proposed BIS-11 subscales (i.e., motor, attention, and nonplanning impulsiveness) lack psychometric stability and have not undergone sufficient psychometric evaluation for use with young adolescents [32, 33]. Thus, we evaluated the latent structure of the BIS-11 in our data prior to conducting statistical analyses (see Appendix for a description of these analyses). Consistent with a large, recent factor analytic study of the BIS-11 in a sample of adults [33], two subscales emerged (i.e., the brief BIS) which reflected impulsive behaviors (e.g., “I say things without thinking”) and impaired self-regulation/planning (e.g., “I plan tasks carefully” (reverse coded)). These subscales evidenced adequate reliability and the distribution of scores for each subscale, which ranged from 4 to 16, approximated a normal distribution (impulsive behaviors: Cronbach's *α* = .74, mean = 7.91 [SD = 2.37]; impaired self-regulation: Cronbach's *α* = .71, mean = 7.89 [SD = 2.52]). The two subscales were correlated moderately (*r* = .48), indicating that behavioral impulsivity and deficits in self-regulation are related domains of impulsivity. However, further examination of the data based on median split demonstrated that there was considerable individual variability in scores on these related, yet distinct, subscales (median for each subscale = 8.00; see Table 1), with 30.8% of participants evidencing high scores on one subscale and low scores on the other.

### 2.3. Program Design Measures

*Type of Prizes.* Students reported on “what types of prizes would make them interested in participating.” Response options included cash, giftcards, mp3 players, cell phones, and other. Students could check as many options as applied. Students selecting “other” were asked to describe the alternative prize. After reviewing all responses (including write-ins), two primary prize types emerged: cash and electronics (e.g., iPods, video games). We created two binary variables reflecting cash and electronics.

*Monetary Value of Prizes.* To determine the minimum monetary value of the prize needed to motivate program interest, students were asked the following question: “What is the smallest prize that would make you interested in participating and staying tobacco-free for the rest of the school year?” Answer choices were $25, $25–50, $50–$75, $75–$100, and $100+.

*Frequency of Rewards.* To determine an ideal reinforcement schedule, students were asked the following question, “How often should the contest be run?” Response options included monthly, every two months, every grading period, every semester, and once a year.

### 2.4. Data Analytic Plan

We conducted data analyses using MPLUS 7. Data were double entered and cleaned. Random spot checks of the original surveys ensured data accuracy. We employed multiple regression to evaluate the extent to which students' interest in program participation was influenced by intrapersonal factors (i.e., gender, age, race/ethnicity, smoking risk status, and impulsivity) and aspects of the incentive-based program design (i.e., prize type, prize value, and reward frequency). We treated the following as categorical predictor variables: gender, race/ethnicity, smoking risk status, and prize type. We included age, impulsivity, prize value, and frequency of reward as continuous predictors. 18.0% of students were missing data on one or more central study variables (at random), so missing data were handled using full information maximum likelihood (FIML).

## 3. Results

The regression model including all variables accounted for 11.2% of the variance in program interest (see Table 2). Younger students, students of non-Caucasian descent, those who self-reported higher levels of engagement in impulsive behavior, and those who reported higher levels of self-regulation were more interested in participating in the program than their respective counterparts. Program interest was also influenced by several program design features. Specifically, higher levels of interest were associated with offering inexpensive rewards more frequently and with offering electronics like video games as rewards. Program interest was not influenced significantly by gender, smoking status, or offering cash incentives.

## 4. Discussion

Our results suggest that the majority of middle school students (61.8%) reported interest in participating in an incentive-based program designed to encourage a smoke-free lifestyle. The program appealed to male and female students. Furthermore, the program appealed to students irrespective of smoking status, suggesting that it has the potential to target both smoking prevention and cessation among middle school students.

Although most students expressed program interest, several variables significantly influenced students' interest level. As anticipated, younger students were more interested than older students. As such, the proposed incentive-based program may be most impactful among younger students, which is encouraging given that the peak ages of cigarette experimentation are 11 to 13 years [34]. The program also was of greater interest to students who were not of Caucasian descent. With regard to impulsivity, students who reported higher levels of engagement in impulsive behavior were more likely to express interest in the program, consistent with prior research indicating that impulsive adolescents may be especially sensitive to reward in the context of smoking cessation programs [20]. Students reporting higher levels of impaired self-regulation/planning were less interested in participating relative to students with higher levels of self-regulation/planning. Students with deficits in planning and concentration may have difficulty appreciating the full range of benefits of participating in an incentive-based program, including the possibility of winning a prize and the inherent health benefits associated with not smoking. However, these students also may optimally benefit from incentive-based programs, as incentives are thought to enhance one's sense of self-control [26]. These students may also perceive participation as requiring an inordinate amount of effort and, therefore, report being uninterested. To maximize participation, making participation as effortless as possible may be key. Setting the optimal default to automatic participation (with the option to opt out) may be superior to providing standard, voluntary enrollment with the option to opt in (i.e., default nonparticipation).

Regarding program design, less expensive prizes offered monthly sufficiently motivated student interest, suggesting that implementing an incentive-based program needs not be costly. Consistent with their tech-savvy moniker “the iGeneration” [35], students' interest was enhanced by offering rewards like video games.

While the current study provides novel information about middle school students' interest in participating in an incentive-based tobacco prevention/intervention program, several limitations merit note. Program interest was assessed using a single question and all data were self-reported. It is worth noting, however, that while reliance on self-reported smoking history introduced the potential for misreporting, our smoking estimates were consistent with national estimates [3]. Our data are also limited in generalizability because they were collected from a small sample of schools in Connecticut. Students from different school environments may have different perspectives on school-based smoking prevention and cessation programs. Further, the small number of lifetime smokers (*n* = 43) limited our statistical power to detect effects specifically within this group. As such, we relied on a broader conceptualization of smoking risk in the current study [36]. Finally, the intrapersonal and program design variables included in the current analyses accounted for only 11.2% of the variance in program interest. An important goal of future work will be to identify a range of other variables (i.e., family, parental, and peer influences) that account for additional variance in program interest and/or help to explain the relationships observed in the current study. For example, socioeconomic status, which was not assessed in the current study, may help to account for the fact that offering electronics as rewards predicted program interest while offering cash rewards did not; it is possible, for instance, that adolescents of lower socioeconomic status may need to put cash rewards toward paying for necessities in the home.

## 5. Conclusions

The current study provides formative data that can inform the development of maximally effective smoking cessation/prevention programs for middle schools that complement existing tobacco education by behaviorally reinforcing a tobacco-free lifestyle. Importantly, students reported strong interest in a cost-effective program irrespective of smoking status. Our data suggest that targeting younger middle school students and offering monthly rewards may maximize program impact. To help boost participation, especially among students with deficits in self-control/planning, automatic enrollment of all students in the program (with the option to opt out) may prove to be a superior voluntary approach to automatic nonenrollment. Adjusting the optimal default to participation may seem unconventional, but drastic measures are warranted given the exorbitant social, economic, and health costs associated with cigarette smoking.

## Figures and Tables

**Table 1:** Sample characteristics based on median split of behavioral impulsivity and deficits in self-regulation.

Behavioral impulsivity	Deficits in self-regulation	% of sample	Behavioral impulsivity mean (SD)	Deficits in self-regulation mean (SD)
High	High	37.6	9.84 (1.76)	10.09 (1.71)
High	Low	16.5	9.18 (1.51)	6.06 (1.00)
Low	High	14.3	6.24 (0.90)	9.29 (1.61)
Low	Low	31.6	5.70 (1.06)	5.59 (1.13)

*Note*. Medians for behavioral impulsivity and deficits in self-regulation = 8.00.

**Table 2:** Predictors of middle school students' interest in program participation.

	*B*	Std. error	*β*
Gender	0.03	0.07	0.01
Age	−0.16∗∗∗	0.04	−0.15
Race	−0.20∗	0.08	−0.08
Cigarette risk status	−0.13	0.07	−0.06
Brief BIS impulsive behavior	0.04∗	0.02	0.08
Brief BIS poor self-regulation	−0.07∗∗∗	0.02	−0.15
Contest frequency	0.14∗∗∗	0.03	0.17
Prize value	−0.07∗∗	0.02	−0.10
Electronic rewards	0.18∗∗	0.07	0.08
Cash rewards	0.03	0.13	0.01

*Note.*  ∗∗∗*P* < .001, ∗∗*P* < .01, ∗*P* < .05. BIS: Barratt impulsiveness scale. Reference groups are gender (female), race (Caucasian), cigarette risk status (low risk nonsmokers), contest frequency (once per year), prize value (<$50), electronic rewards (electronics), and cash rewards (cash).

## Appendix

We used Mplus7 to fit three confirmatory factor analytic models in which we evaluated the original 3-factor solution [31], a single factor solution [32], and a two-factor solution recently identified across several independent samples including undergraduates, adults seeking smoking cessation treatment, and adults participating in alcohol research [33]. For each model, we specified robust maximum likelihood estimation given its robustness to nonnormality and its ability to produce model fit indices (e.g., CFI, RMSEA). To account for theorized correlations among latent factors in the 2- and 3-factor models, we specified an oblique rotation (i.e., CF-Varimax (oblique)). We handled missing data using full information maximum likelihood. We used the following statistical cutoffs to determine adequacy of model fit: Bentler's comparative fit (CFI)>.90 [37], root mean square error of approximation (RMSEA)<.07 [38], and standardized root mean square residual (SRMR)<.05 [39]. Based on these criteria, neither the original 3-factor model (CFI = .63; RMSEA = .07, SRMR = .08) nor the single-factor, brief version of the BIS-11, fit the data (CFI = .89, RMSEA = .084, SRMR = .05). The 8-item, 2-factor structure of the brief BIS [33] reflecting behavioral impulsivity and poor self-regulation fits the data well (CFI = .95, RMSEA = .06, SRMR = .04).
